# Stereoscopic three-dimensional visualization applied to multimodal brain images: clinical applications and a functional connectivity atlas

**DOI:** 10.3389/fnins.2014.00328

**Published:** 2014-11-06

**Authors:** Gonzalo M. Rojas, Marcelo Gálvez, Natan Vega Potler, R. Cameron Craddock, Daniel S. Margulies, F. Xavier Castellanos, Michael P. Milham

**Affiliations:** ^1^Laboratory for Advanced Medical Image Processing, Department of Radiology, Clínica las CondesSantiago, Chile; ^2^Advanced Epilepsy Center, Clínica las CondesSantiago, Chile; ^3^Department of Radiology, Clínica las CondesSantiago, Chile; ^4^The Child Study Center, Department of Child and Adolescent Psychiatry, New York University Langone Medical CenterNew York, NY, USA; ^5^Center for the Developing Brain, Child Mind InstituteNew York, NY, USA; ^6^Center for Brain Imaging and Neuromodulation, Nathan Kline Institute for Psychiatric ResearchOrangeburg, NY, USA; ^7^Max Planck Institute for Human Cognitive and Brain SciencesLeipzig, Germany; ^8^Division of Child and Adolescent Psychiatry Research, Nathan Kline Institute for Psychiatric ResearchOrangeburg, NY, USA

**Keywords:** stereoscopic, anaglyph, intrinsic connectivity networks, atlas, functional connectivity, 3D visualization, functional connectivity atlas

## Abstract

Effective visualization is central to the exploration and comprehension of brain imaging data. While MRI data are acquired in three-dimensional space, the methods for visualizing such data have rarely taken advantage of three-dimensional stereoscopic technologies. We present here results of stereoscopic visualization of clinical data, as well as an atlas of whole-brain functional connectivity. In comparison with traditional 3D rendering techniques, we demonstrate the utility of stereoscopic visualizations to provide an intuitive description of the exact location and the relative sizes of various brain landmarks, structures and lesions. In the case of resting state fMRI, stereoscopic 3D visualization facilitated comprehension of the anatomical position of complex large-scale functional connectivity patterns. Overall, stereoscopic visualization improves the intuitive visual comprehension of image contents, and brings increased dimensionality to visualization of traditional MRI data, as well as patterns of functional connectivity.

## Introduction

Visualization is critical to understanding and reporting neuroimaging data. The complexities of brain geometry and its variation from one individual to the next, combined with the increasing number of imaging modalities (MRI, PET, SPECT, fMRI, DTI) and representations (e.g., parcellations, voxels, vertices) required to fully characterize its structure and function, make visualization a formidable challenge. Arguably, the increased emphasis on patterns of brain connectivity has ushered in a new level of challenge due to the vast complexity of the connectome. Classical methods for viewing clinical and research neuroimaging data, such as print media or radiological film, reduce the three-dimensional (3D) structure of the brain to two-dimensional (2D) representations, resulting in significant loss of information. However, these 2D visualizations are not mandated by technical limitations or fundamental limits of human perception. By taking advantage of stereoscopy—an approach for the visualization of data in 3D that dates back nearly two centuries—the higher dimensional structure of neuroimaging data can be faithfully reproduced.

In 1838 the English physicist Sir Charles Wheatstone first defined the concept of stereopsis as the perception of depth that results from slight differences in the visual projection of the world onto the two retinas due to their distinct anatomical positions (Wheatstone, 1838). This phenomenon is commonly referred to as binocular disparity, retinal disparity, or retinal rivalry (Lipton, 1982). Later in the same year, Sir Charles Wheatstone created a device called a “stereoscope,” which provides each eye with a distinct picture. Using two angled mirrors, he demonstrated the human brain's tendency to bring together the two images in a manner that yields perception of a 3D visualization (Wheatstone, 1842). Finally, Wilhelm Rollmann, a physicist, built upon this concept to devise the anaglyph, which employs red and green lenses to create a 3D visualization by showing different pictures filtered by red and green lenses to each eye (Rollmann, 1853).

In short, *stereoscopy* refers to the process of creating or enhancing the illusion of depth in an image by presenting two offset images separately to the left and right eyes of the viewer. These images are combined by the visual processing system in the human brain to give the perception of 3D depth. Currently there are three stereoscopic approaches to 3D display, which include:

1. Stereograms, which can be:
  - spatially directed or filtered with glasses (e.g., stereoscopes, Wheatstone, 1842; Brewster, 1856, anaglyphs, Rollmann, 1853, polarized images, Zone, 2007)
  - temporally filtered with glasses (e.g., shutter glasses, Turner and Hellbaum, 1986; Asthana and Sinha, 2005)
  - perceptually delayed with glasses (e.g., Pulfrich effect, Pulfrich, 1922; Morgan and Thompson, 1975; McAllister, 2002)
  - spatially directed or filtered without glasses, “autostereograms” (Julesz, 1960, 1964) (e.g., lenticular views, McAllister, 2002, polarizing filters and half-silvered mirrors, coarse gratings, Tyler, 1974, louvers)
2. Slice-stack methods (non-occluding temporal reconstruction, e.g., varifocal mirrors, Traub, 1967; Rawson, 1969, rotating helical surfaces, beam-excited fluorescing gases).
3. Wavefront reconstruction methods (e.g., holograms, Gabor, 1948; Denisyuk, 1962; Benton, 1969; Phillips and Porter, 1976, numerical holograms, Schnars and Jüptner, 2005).

For anaglyphs, the viewer wears eyeglasses with two different color lenses (usually chromatically opposite), such as red and cyan. The picture contains two differently colored images that have a slight offset, which is seen by each eye when filtered by the colored lenses. Such anaglyphs can be viewed in several types of media: computer screens (for example, Internet web pages), cinema, TV (videogames, DVD), digital projectors, or print. Different color combinations can be used for anaglyph eyeglasses. For example (generally in left-right eye order; Ribas et al., 2001; Hawkins, 2002): red-green (pure colors), red-blue (pure colors), or red-cyan (pure red, pure green+blue). In the first two cases the color rendering is monochrome, and in the third it is full color (good color perception of green and blue, poor perception of red).

Over the years, various applications of red-cyan anaglyph 3D method have emerged for the purposes of anatomical visualization in medicine. For example, Hirsch and Kramer used cyan-red anaglyphs to create a paper and CD-ROM based human brain atlas, entitled “Neuroanatomy: 3D-Stereoscopic Atlas of the Human Brain” (Hirsch and Kramer, 1999). Providing 173 illustrations of interactive and rotatable 3D models, this work demonstrated the value of 3D stereoscopy for visualizing the human brain—especially when augmented with modern-day computing capabilities. Another example is provided by Guilherme Carvalhal Ribas (Ribas et al., 2001) who devised a 3D anaglyph-based approach for visualizing anatomical and surgical images obtained by cameras affixed to a surgical microscope; this resource was intended for both teaching and generating surgical reports.

Here we introduce the application of anaglyph-based 3D stereoscopy to visualization of data obtained from various MR-based imaging modalities. First, we illustrate the utility of anaglyph-based viewing of MRI data spanning commonly used imaging modalities (i.e., morphometry, diffusion imaging, and functional MRI), including clinical data (e.g., neoplasms). Then, we provide illustrations and descriptions of an interactive full brain functional connectivity atlas we developed using 3D stereoscopic visualization techniques. The atlas uses seed-based correlation analyses and contains illustrations for the voxel-wise connectivity maps associated with each of the 200 functional regions in the brain recently identified by Craddock et al. (2012) using data-driven cluster analysis approaches. While various parcellation schemes are available for the brain, this approach was selected due to its emphasis on the delineation of functional (rather than structural) regions as the basis for functional connectivity regions-of-interest (ROIs).

## Materials and methods

### Morphometric illustrations for clinical cases

Morphometric studies for eight clinical cases are included in the present work for the purpose of demonstration, after receiving express written permission from the patients. Stereoscopic illustrations were generated for each of the cases using 3D Slicer version 3.6.3 anaglyph stereo option (Brigham & Women's Hospital, Boston, Massachusetts, USA, www.slicer.org; Gering et al., 1999; Pieper et al., 2004, 2006). The imaging parameters used for the clinical cases are provided below.

#### Clinical cases P1-P4

For clinical cases P1-P4 (see **Table 2** for clinical data), a high resolution T1-weighted magnetization prepared gradient echo (MPRAGE) sequence (sagittal images, 256 × 256 × 160, 1 mm^3^ isotropic spatial resolution, *TI* = 1100 ms, *TR* = 1870 ms, *TE* = 3.25 ms, flip angle = 15°) was obtained using a Siemens-Avanto 1.5T MRI scanner (Siemens, Erlangen, Germany). The segmentation and volumetric studies were processed using FreeSurfer 4.4.0 (Dale et al., 1999; Fischl et al., 1999; Fischl and Dale, 2000).

#### Clinical case P5

**Figure 1:**
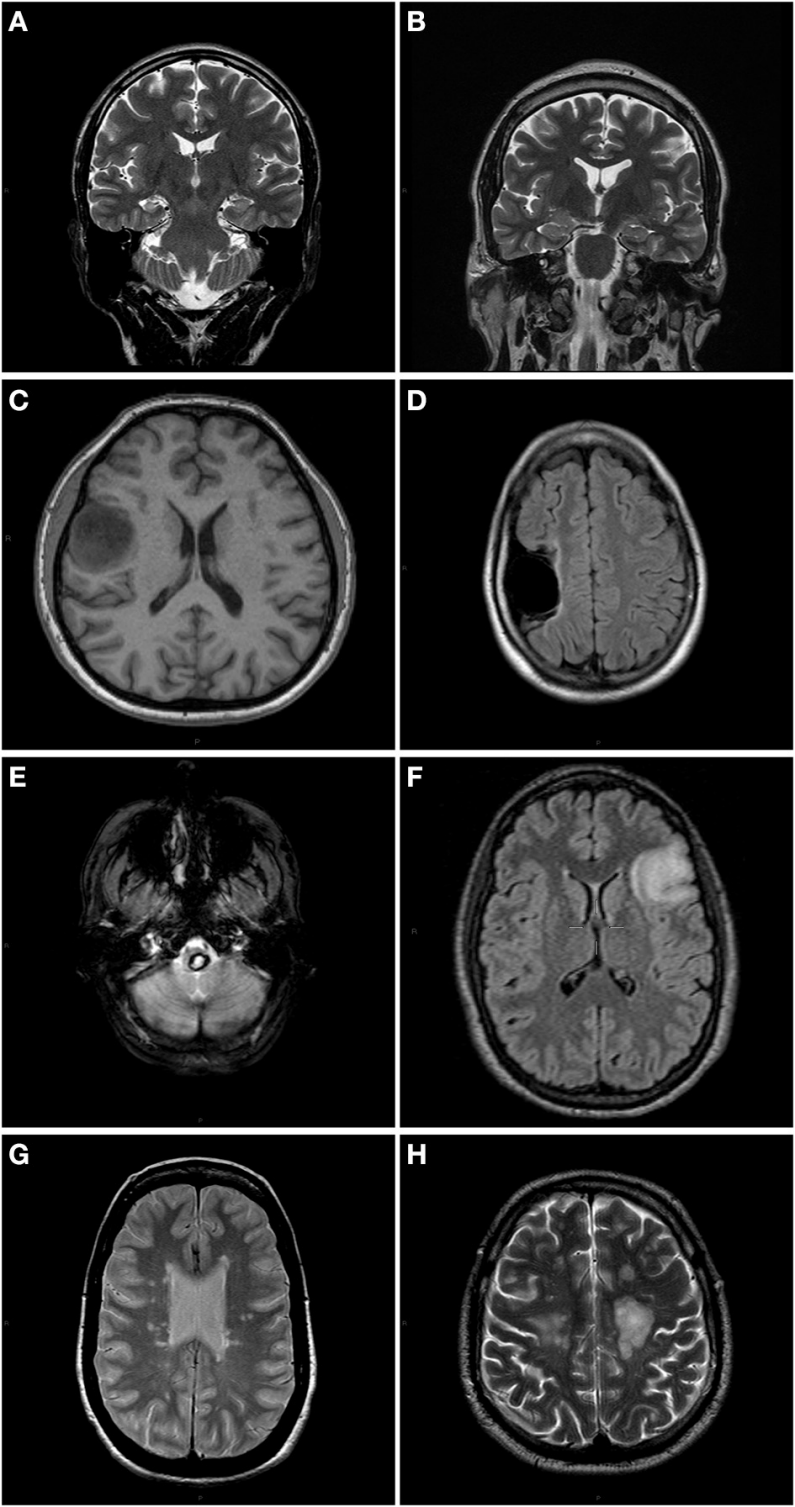
**Anatomical slices of each patient showing different type of lesions**. **(A)** P1: T2 weighted coronal slice. The right hippocampus volume is reduced. **(B)** P2: T2 weighted coronal slice. Slight decrease in right hippocampus volume. **(C)** P3: T1 weighted axial slice showing right precentral tumor. **(D)** P4: T2- FLAIR axial image showing a right temporal porencephalic cyst. **(E)** P5: T2 weighted axial slice of a patient with a medulla oblongata cavernoma in brainstem. **(F)** P6: T2-FLAIR of a left frontal glioma patient. **(G)** P7: PD axial slice of a MS patient. **(H)** P8: T2 weighted axial slice of a MS patient.

For case P5, a 63 year-old female patient with a medulla oblongata cavernoma, a diffusion tensor imaging (DTI) sequence (single-shot diffusion-weighted spin-echo EPI sequence, *TR* = 7100 ms, *TE* = 96 ms, matrix = 116 × 116, FOV = 230 × 230 mm, slice thickness 2.2 mm, gap = 0.8 mm, 50 contiguous sections, *b* = 1000 s/mm^2^, 30 non-collinear directions), a high resolution isotropic T1-weighted magnetization prepared gradient echo sequence was acquired with the same parameters as cases P1-P4, and post gadolinium isotropic T1-weighted images (T1-GD, sagittal images, 256 × 256 × 160, 1 mm^3^ isotropic spatial resolution, *TI* = 1100 ms, *TR* = 2060 ms, *TE* = 3.25 ms, flip angle = 15°) were collected. See standard T2-weighted image in Figure 1E. The tractography was processed using 3D Slicer 3.6.3 software. The T1-GD, T1 and DTI were coregistered with the 3D Slicer linear registration algorithm, and the cavernoma was segmented using a T1-GD sequence with a simple region-growing algorithm, which was then used to create a 3D model of the cavernoma using the *Model Maker* module of 3D Slicer.

#### Clinical case P6

For case P6, a 24 year-old female patient with a left frontal glioma, a standard fMRI block-design language generation task was used to activate Broca's area in the left posterior inferior frontal gyrus (Binder, 2006). Blood-oxygenation level-dependent (BOLD) signals were measured with a T2^*^-weighted echo-planar imaging (EPI) sequence (*TR* = 2890 ms, *TE* = 50 ms, flip angle = 90°, FOV = 192 × 192 mm, in-plane resolution = 64 × 64 pixels or 3 × 3 mm). Twenty interlaced axial slices, with 5.0 mm thickness (gap = 1.25 mm) were acquired. Each acquisition had 55 scans including 5 dummy scans that were discarded from analysis. A standard 3D structural sagittal T1-weighted MPRAGE image used the acquisition parameters described previously. See standard T2-Flair image in Figure 1F. The fMRI was processed using FSL 4.1.8 software package (Smith et al., 2004; Woolrich et al., 2009). A 3D sagittal Fluid Attenuated Inversion Recovery (FLAIR) image (*TE* = 354 ms, *TR* = 7000 ms, flip angle = 180°, FOV = 294 × 212, in-plane resolution = 256 × 184 pixels or 1.15 × 1.15 mm) was used to segment the glioma using a simple region growing algorithm and then to create a 3D model of the tumor using the *Model Maker* module of 3D Slicer software package.

#### Clinical cases P7-P8

For two individuals with multiple sclerosis (P7: a 37 year-old female, P8: a 42 year-old male), the imaging protocols included 3D sagittal FLAIR images (same parameters as above) and a 3D structural sagittal T1-weighted MPRAGE image using the acquisition parameters described previously (Figures 1G,H). Multiple sclerosis (MS) lesion segmentation and lesion load was done using Toads-Cruise 2010 R2a Medical Image Processing, Analysis, and Visualization (MIPAV) software (http://mipav.cit.nih.gov) plug in (McAuliffe et al., 2001; Shiee et al., 2010). The *Model Maker* module of 3D Slicer software package was used to create the 3D model of the MS lesions.

### Resting state fMRI based functional connectivity illustrations

#### Participant data

Resting state fMRI scans of 45 healthy volunteers (age range = 18–27, mean age = 21.0 ± 2.2, 3T MRI) from the Cambridge-Buckner dataset of the 1000 Functional Connectomes Project (www.nitrc.org/projects/fcon_1000; Biswal et al., 2010) were used to illustrate the utility of anaglyphs for visualizing intrinsic connectivity networks (ICNs).

#### Image preprocessing

Data processing was performed using Analysis of Functional NeuroImaging (AFNI; http://afni.nimh.nih.gov/afni; Cox, 1996) and fMRIB Software Library (FSL; www.fmrib.ox.ac.uk). Image preprocessing consisted of slice time correction for interleaved acquisitions; 3D motion correction with Fourier interpolation; despiking (detection and compression of extreme time series outliers); spatial smoothing using a 6 mm FWHM Gaussian kernel; mean-based intensity normalization of all volumes by the same factor; temporal bandpass filtering (0.009–0.1 Hz); and linear and quadratic detrending.

Linear registration of high-resolution structural images to the MNI152 template was carried out using the FSL tool *FLIRT* (Jenkinson and Smith, 2001; Jenkinson et al., 2002). This transformation was then refined using *FNIRT* non-linear registration (Andersson et al., 2007a,b). Linear registration of each participant's functional time series to the high-resolution structural image was performed using *FLIRT*. This functional-to-anatomical co-registration was improved by intermediate registration to a low-resolution image and b0 unwarping.

To control for the effects of physiological processes (such as fluctuations related to cardiac and respiratory cycles) and motion, we regressed each participant's 4-D preprocessed volume on nine predictors that modeled nuisance signals from white matter, CSF, the global signal, and six motion parameters. Each participant's resultant 4-D residuals volume was spatially normalized by applying the previously computed transformation to MNI152 standard space with 2 mm^3^ resolution.

#### Default network

After image preprocessing described in the previous section, 6 mm radius spherical seeds located in medial prefrontal cortex (MPF, MNI coordinates: −1, 47, −4), posterior cingulate/precuneus (PCC, −5, −49, 40) and lateral parietal cortex (LP, −45, −67, 36) were used to generate participant-level default network maps (Fox et al., 2005; Mennes et al., 2010). All group maps were corrected for multiple comparisons using Gaussian random field theory (min *Z* > 2.3; cluster significance: *p* < 0.05, corrected).

The image fusion and stereoscopic 3D images of each case were created using 3D Slicer 3.6.3 software (Gering et al., 1999; Pieper et al., 2004, 2006). 3D Slicer implemented the red-cyan anaglyph method for producing stereoscopic 3D images.

#### Craddock functional connectivity atlas

A 3D stereoscopic full brain functional connectivity atlas was created using an atlas published by Craddock et al. (2012). Using 3D Slicer 3.6.3 and the 200 ROI version of the Craddock atlas, 200 grayscale surface models were created using a z-stat threshold > 2.3, and each surface model was processed with a surface decimation algorithm (Target reduction = 0.2), smoothed with the Taubin algorithm (5 iterations, Taubin passband = 0.1) and without surface normals. The surface model of the 200 ROIs of the Craddock parcellation was created using the Model Maker 3D Slicer module with 10 smoothing iterations, Sinc type smoothing, and 0.25 decimal percentage of target reduction in the number of polygons.

For improved visualization of the functional connectivity networks and their relative anatomical position, the surface model of five subcortical anatomical structures (corpus callosum, bilateral caudate, pallidum, putamen, thalamus, and hippocampus) were included in ICN visualizations. These surfaces were created with 3D Slicer using the segmentation computed with FreeSurfer v. 5.1.

#### Evaluation of visualization techniques

Twenty-one 3D images first and subsequently the same stereoscopic versions were shown to a group of 14 healthcare professionals (8 pediatric and adult neurologists, 1 neuroradiologist, 2 neurosurgeons, 1 radiologist, and 2 nurses; 30–52 years old). For each image, they were asked about the utility of each version of the image (3D, stereoscopic) in the assessment of a distinct brain feature. The questionnaire is included in Table 1, and each question was answered on a Likert scale from 0 (not at all) to 5 (perfectly).

**Table 1:** **Questions used for 2D and Stereoscopic versions of some of the images included in this paper to test stereoscopic benefits**.

**Images**	**How well, in a scale 0 (not at all)—5 (perfectly)…**	**Answer 0 (not at all)—5 (perfectly)**
1	Is it possible to estimate the length of each hippocampus?	
2	Is it possible to estimate the position of an hippocampus relative to the other?	
3	Is it possible to estimate the length of each hippocampus?	
4	Is it possible to estimate the depth of the tumor?	
5	Is it possible to estimate the depth of the tumor?	
6	Is it possible to estimate the depth of the tumor?	
7	Is it possible to estimate the depth of the tumor?	
8	Is it possible to estimate the relative position of the tumor to the tracts?	
9	Is the relative position of Broca's area with respect to the tumor viewed using anaglyph?	
10	Is the relative position of Broca's area with respect to the tumor viewed using anaglyph?	
11	Is it possible to determine the distance between Broca's area and the tumor?	
12	Is it possible to determine the relative position of a MS lesion with respect to another?	
13	Is it possible to determine the relative position of a MS lesion with respect to another?	
14	Is it possible to determine the relative position of a MS lesion with respect to another?	
15	Is it possible to determine the relative position of each area of the functional network (Default Mode Network) with respect to another?	
16	Is it possible to determine the relative position of each area of the functional network (Default Mode Network) with respect to another?	
17	Is it possible to determine the relative position of each area of the functional network (Default Mode Network) with respect to another?	
18	Is it possible to determine the relative position of the functional network with respect to the hippocampus?	
19	Is it possible to determine the relative position of the different regions of the functional network?	

**Figure 2:**
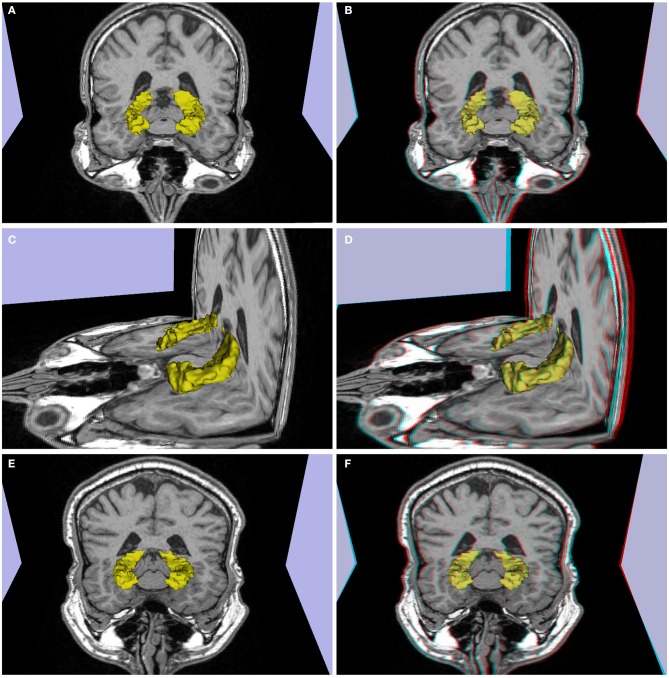
**(A–D)** Patient P1 (left temporal lobe refractory epilepsy, decrease in volume and signal hyperintensity of the right hippocampus consistent with mesial temporal sclerosis in a 26 year-old). **(E,F)** Patient P2 (single epileptic seizure, arterial hypertension, dislipidemia, 44 years old), slight decrease in right hippocampus volume. **(A,C,E)** 3d images, **(B,D,F)** stereoscopic (anaglyph) 3D version that must be viewed using red-cyan anaglyph glasses.

## Results

### Clinical illustrations

To demonstrate the added value of stereoscopy in the visualization of structural and functional abnormalities in clinical patients we created anaglyph-based 3D images for a series of magnetic resonance studies. Below we discuss highlights from representative cases of commonly found abnormalities (common and anaglyphic 3D images are provided for each case to enable comparison. Anaglyph images must be viewed using red-cyan anaglyph glasses from computer screen or printed matter). See Table 2 for clinical descriptions.

**Table 2:** **Data summary for each patient**.

**Patient**	**Demographic information**	**Clinical data**	**Image sequences**	**Figure numbers**
P1	26 year-old, male	Left temporal lobe treatment-refractory epilepsy, right hippocampus volume reduction, mesial temporal sclerosis	MPRAGE	Figures 1A, 2A–D
P2	44 year-old, male	Single epileptic seizure, arterial hypertension, dyslipidemia, slight decrease in right hippocampus volume	MPRAGE	Figures 1B, 2E,F
P3	36 year-old, female	Epilepsy secondary to right precentral tumor	MPRAGE	Figures 1C, 3A–D, 4A,B
P4	25 year-old, female	Epilepsy secondary to a right temporal porencephalic cyst	MPRAGE	Figures 1D, 5A–D
P5	63 year-old, female	Medulla oblongata cavernoma	MPRAGE, T1-GD, DTI	Figures 1E, 6A,B
P6	24 year-old, female	Left frontal glioma	MPRAGE, EPI	Figures 1F, 7A–F
P7	37 year-old, female	Multiple sclerosis	MPRAGE, FLAIR	Figures 1G, 8A,B
P8	42 year-old, male	Multiple sclerosis	MPRAGE, FLAIR	Figures 1H, 8C–F

#### Mass lesions

**Figure 3:**
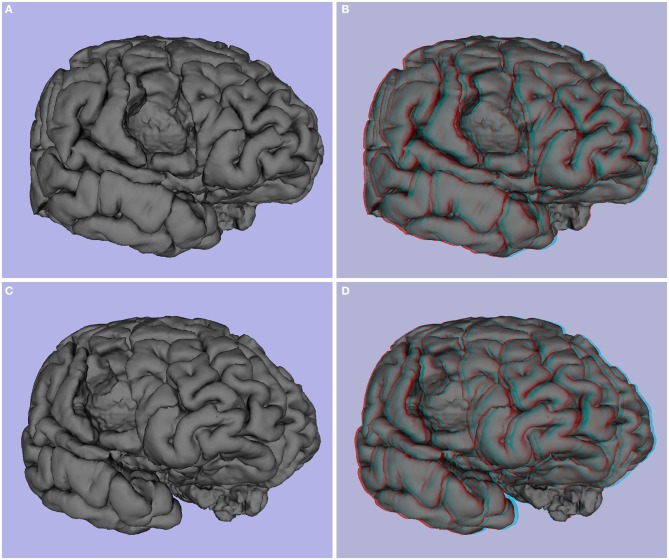
**Patient P3 (epilepsy secondary to right precentral tumor, 36 years old)**. **(A,C)** 3D image, **(B,D)** and stereoscopic (anaglyph) 3D of pial surface.

**Figure 4:**
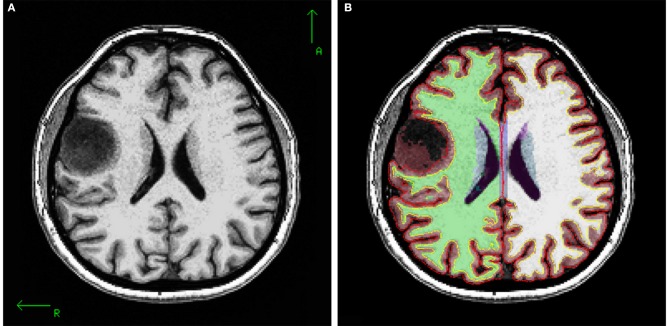
**(A)** T1 axial slice of P3 showing right hemisphere precentral tumor, **(B)** segmentation, white matter and pial surface of same slice. The tumor was segmented as an “unknown” tissue, and white and pial surfaces encircle the tumor.

One of the most obvious potential applications of stereoscopy is the visualization of mass lesions, such as tumors and cysts. To demonstrate this point, we first generated common and anaglyphic 3D visualizations from the MPRAGE data for patient P3, a 36 year-old female with history of epilepsy secondary to right precentral tumor (see Figures 3, 4, and Supplementary Video 1). FreeSurfer segmentation identified the tumor as an “unknown” tissue, and the resultant mesh of the right hemisphere was notable for a hole in the pial surface (see Figures 4A,B). While both common and anaglyph 3D images show the tumor in detail, as well as its anatomical position, the anaglyph version is notably more realistic, as it more effectively conveys the depth of the tumor.

**Figure 5:**
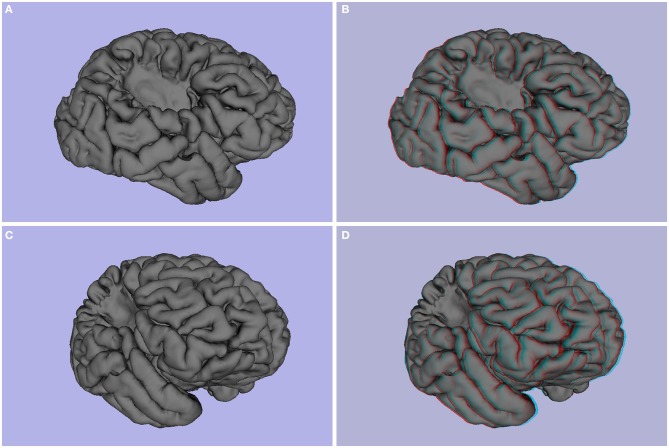
**Patient P4 (epilepsy secondary to porencephalic cyst, female, 25 years old)**. **(A,C)** 3D images, **(B,D)** stereoscopic (anaglyph) 3D of pial surface.

Next, we generated common and anaglyphic 3D images from the MPRAGE data for patient P4, who was diagnosed with epilepsy secondary to a right temporal porencephalic cyst. As is notable in Figure 5, the cyst, appearing as a large hole in the right temporal lobe of the brain, is better conveyed with stereoscopic visualization. This advantage is due to the more effective representation of the depth of the hole produced by the cyst, as well as the relative geometry of the adjacent gyrus and sulcus, which further enhances the realism of the image.

**Figure 6:**
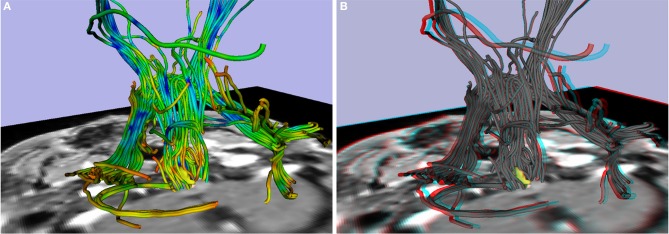
**Patient P5 (medulla oblongata cavernoma, 63 years old)**. Tractography with cavernoma in yellow: **(A)** 3D image, **(B)** stereoscopic (anaglyph) 3D image.

Beyond visualization of MPRAGE-based morphometric findings, stereoscopic approaches can be extended to the visualization of diffusion imaging-based findings. In this regard, we provide common and anaglyphic versions of tractography visualizations of patient P5, a 63-year old female with a medulla oblongata cavernoma depicted in yellow in Figure 6. Figure 6A shows a standard presentation of tractography, while Figure 6B (and Supplementary Video 2) is a stereoscopic depiction. The approaches are notably different, with Figure 6A using variations in color related to FA (fractional anisotropy) to convey details; in contrast, Figure 6B depicts the tracts in gray to facilitate stereoscopic viewing (colors need to be removed to prevent any unintended impact on the anaglyph glasses). Despite the lack of color, the stereoscopic image shows the relative position of the tracts with respect to the cavernoma and the different sections of the tracts.

#### Atrophy

Another potential use of stereoscopy is to enhance the ability to visually identify atrophy in brain structures commonly affected by aging, or mesial temporal sclerosis, such as the hippocampus. 3D images appear in Figures 2A,C, and stereoscopic images of P1 appear in Figures 2B,D. P2 related images appear in Figures 2E,F in 3D and stereoscopic versions, respectively. The hippocampus is shown in yellow. The left hippocampus is slightly atrophied and the right shows moderate atrophy. The stereoscopic visualization allows one to perceive the difference in size between both hippocampi and their differences in relative position in a realistic way.

#### White matter lesions

Central to the diagnosis of myelin-related disorders such as multiple sclerosis is the visualization and identification of white matter lesions. To demonstrate the potential utility of stereoscopy, we provide common and anaglyphic 3D illustrations of MPRAGE and FLAIR images from two patients with multiple sclerosis (MS patients P7 and P8; see Figure 8 and Supplementary Video 3). Figures 8A,C,E depict the common “3D” images, and Figures 8B,D,F illustrate the stereoscopic forms. The stereoscopic images show the relative position of the MS lesions between patients, and between lesions and anatomical slices. Figure 8F shows the relative position of each MS lesion: the lesions in the left hemisphere appear closer to the observer, and the lesions in the right hemisphere appear farther away, with the corpus callosum located between them.

**Figure 7:**
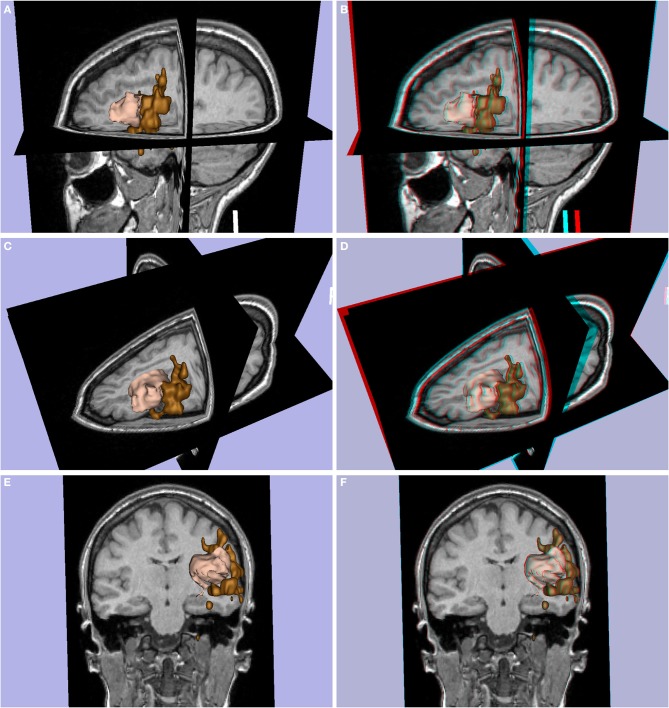
**Patient P6 (left frontal glioma, female, 24 years old)**. Glioma in pink with Broca's area (expressive language fMRI) in brown. **(A,C,E)** 3D images, **(B,D,F)** stereoscopic (anaglyph) 3D version.

**Figure 8:**
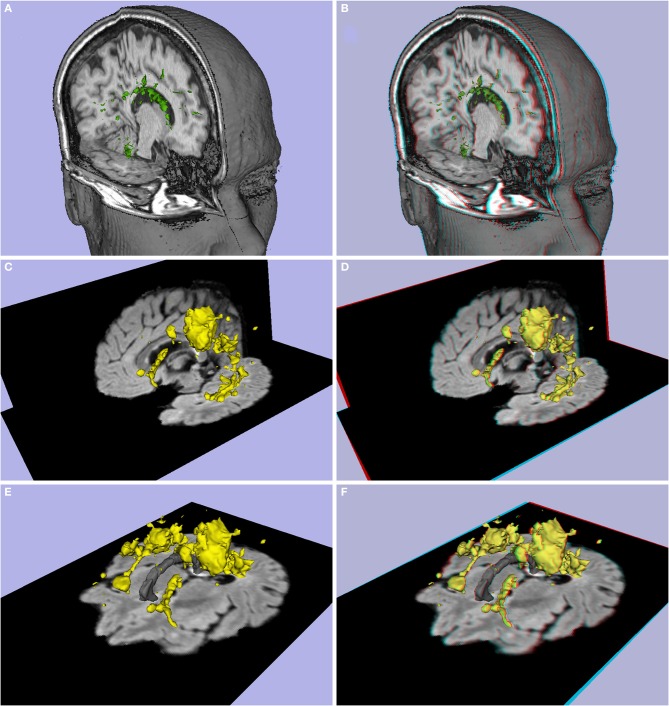
**(A,B)** Lesion load of patient P7 (Multiple sclerosis, difficulty in walking, 37 year-old female), **(C–F)** lesion load of patient P8 (Multiple sclerosis, 42 year-old male). **(A,C,E)** 3D images, **(B,D,F)** stereoscopic (anaglyph) 3D version.

#### Altered functional activation

A final application to clinical examinations is provided by the illustration of altered functional activation in pathologic brain regions. Figure 7 shows the 3D (Figures 7A,C,E) and stereoscopic images (Figures 7B,D,F) of a left frontal glioma in a 24-year old patient (P6) with expressive language fMRI activation. The fMRI activation and glioma are shown in 3D models (pink and brown colors respectively). The models show that the expressive language activation (Broca's area) is in close contact with the frontal glioma.

### Neurotypical illustrations

#### Resting state fMRI based functional connectivity stereoscopic atlas

**Figure 9:**
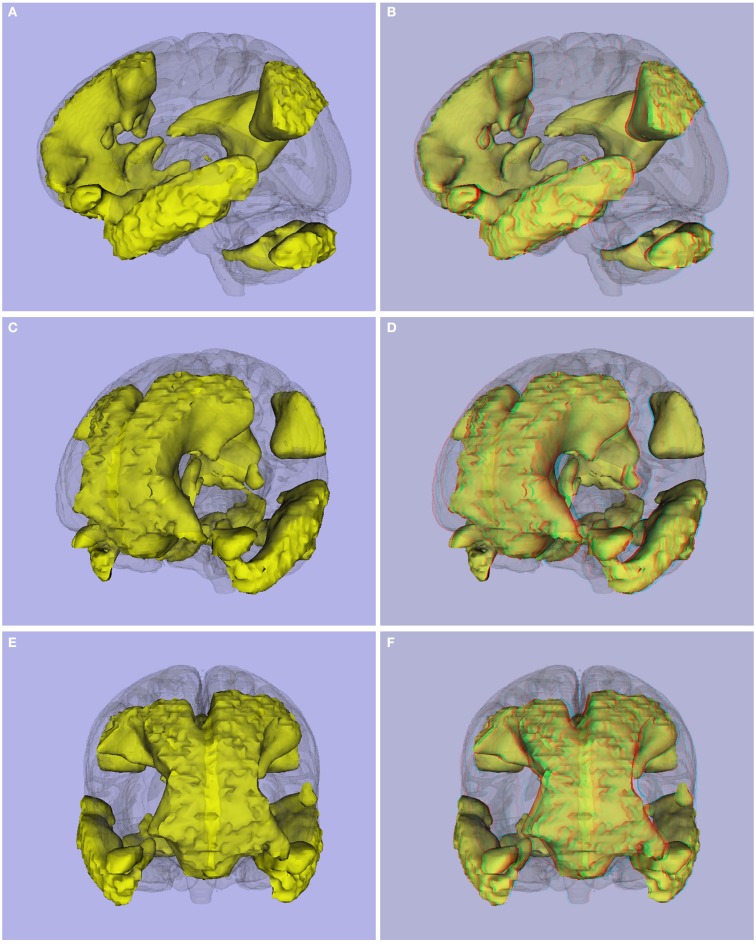
**(A,C,E)** 3D viewing, and **(B,D,F)** stereoscopic (anaglyph) version of Default Mode Network obtained using resting state images from 45 controls.

We selected the default network for our primary demonstration of the utility of stereoscopy in visualizing large-scale functional networks, as it is one of the most commonly examined and widely distributed networks in the brain. As demonstrated in Figure 9 (and Supplementary Video 4), using the anaglyph approach, we can perceive the relative location, morphology and size of key structures in the network (i.e., medial temporal lobe, medial prefrontal cortex, posterior cingulate cortex, precuneus, and parietal cortex; see Figures 9B,D,F, and Supplementary Video 4).

**Figure 10:**
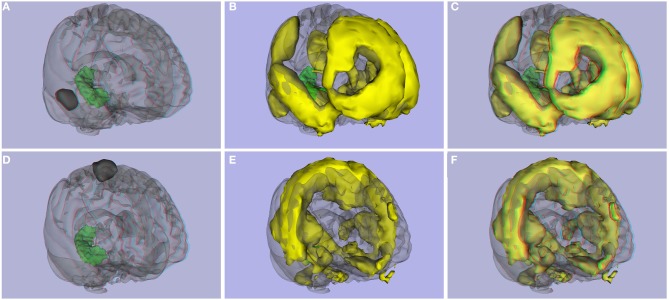
**Functional connectivity atlas**. **(A)** ROI 2 and right hippocampus, **(B)** 3D image, and **(C)** stereoscopic (anaglyph) 3D version of functional connectivity network created with ROI 2. **(D)** ROI 126 from the Craddock parcellation atlas and right hippocampus, **(E)** 3D image, and **(F)** stereoscopic (anaglyph) version of functional connectivity network created with ROI 126.

The anaglyph-based approach can be applied to any functional network in the brain. Building upon this strength, we created a functional atlas of the brain (see Figure 10), including the networks for each of the 200 functional units identified in a recent functional-connectivity based parcellation of the brain (Craddock et al., 2012). The complete stereoscopic functional connectivity atlas (Stereoscopic Atlas of Intrinsic Brain Networks; SAIBN) is available at NITRC (www.nitrc.org/projects/saibn/).

Of note, to better visualize the position of a functional network in the brain, a solution used in this paper was the addition of a surface model of subcortical structures (such as hippocampus, amygdala, corpus callosum, 3rd and 4th ventricles, lateral ventricles, putamen, pallidum, caudate, thalamus). The same approach could be used for our functional connectivity atlas to perceive the relative position of a functional connectivity network with respect to the hippocampus or the midline of the corpus callosum, for example.

#### Evaluation of visualization techniques

Answers to questions related to the 3D and stereoscopic images are summarized in Table 3. The mean score for the 19 questions, across the 14 healthcare professionals is 2.5 (range: 1.5–3.3) for 3D images and 4.3 (range: 3.8–4.8) for stereoscopic versions with a standard deviation of 2.6 and 0.9, respectively. Using a Student's *t*-Test (α = 0.05) the difference between 3D and stereoscopic visualization was statistically significant [*t*_(18)_ = 9.88, *p* = 1.07E-08]. Stereoscopic visualizations were consistently ranked higher by raters as indicated by a Fleiss' Kappa = 0.255, *p* < 0.001 (Altman, 1991).

**Table 3:** **Answer to the questions of Table 1**.

**HP**	**1**		**2**		**3**		**4**		**5**		**6**		**7**		**8**		**9**		**10**		**11**		**12**		**13**		**14**	
**Images**	**3D**	**S3D**	**3D**	**S3D**	**3D**	**S3D**	**3D**	**S3D**	**3D**	**S3D**	**3D**	**S3D**	**3D**	**S3D**	**3D**	**S3D**	**3D**	**S3D**	**3D**	**S3D**	**3D**	**S3D**	**3D**	**S3D**	**3D**	**S3D**	**3D**	**S3D**
1	3	4	3	4	2	3	0	5	3	4	3	3	3	4	3	4	3	4	5	5	4	4	3	4	3	4	3	4
2	4	5	0	4	4	3	0	5	3	4	3	3	3	4	2	4	5	4	3	4	2	5	4	5	3	4	2	4
3	2	4	3	4	2	4	0	5	2	3	3	3	3	4	1	4	2	3	4	4	2	4	3	4	3	4	2	4
4	3	5	2	4	0	4	3	5	1	4	0	3	2	4	0	4	2	4	0	5	0	4	0	5	3	5	0	4
5	3	5	3	4	0	4	3	5	2	5	0	4	2	4	0	4	1	4	1	5	0	4	3	5	3	5	0	4
6	3	5	2	5	0	4	3	5	1	4	0	4	2	4	0	5	1	4	1	5	0	4	1	4	1	4	1	4
7	2	5	2	5	0	4	3	5	2	5	0	4	2	4	0	4	1	5	1	4	0	4	1	4	0	4	1	5
8	4	5	4	5	3	3	4	4	3	5	3	5	3	4	3	4	3	4	1	5	4	4	1	5	2	5	1	5
9	5	5	4	4	3	4	4	4	3	3	2	4	3	4	3	3	4	5	4	5	4	4	5	5	4	4	3	4
10	5	5	3	4	3	4	4	4	3	5	3	5	3	4	3	4	4	4	4	5	4	4	5	5	4	4	3	5
11	3	5	2	3	2	4	3	4	1	3	0	5	3	4	3	3	1	3	1	4	2	4	3	5	2	4	1	5
12	3	5	3	5	3	4	5	5	1	4	2	4	3	5	4	5	4	5	4	5	4	5	4	5	2	4	3	5
13	3	5	3	5	3	4	4	5	2	4	2	4	3	5	2	5	3	5	2	5	4	5	1	5	2	4	3	5
14	4	5	4	4	3	4	4	5	3	4	2	4	3	5	4	5	4	5	3	5	4	5	4	5	4	4	3	5
15	3	5	3	5	3	4	4	5	3	5	1	4	3	4	2	5	4	5	3	4	4	5	1	4	3	5	3	4
16	3	5	3	5	3	4	3	5	3	4	3	5	3	5	2	5	4	5	3	5	4	5	3	5	4	5	3	4
17	3	5	3	5	3	4	4	5	2	4	1	5	3	5	2	5	4	5	3	4	4	5	3	4	3	5	3	4
18	3	4	3	5	2	4	3	4	1	3	1	4	3	4	4	5	3	5	2	4	3	4	3	4	3	4	3	4
19	3	4	3	4	2	4	2	3	1	3	0	4	3	4	4	4	3	4	2	2	2	4	0	5	2	4	3	5
Mean	3.3	4.8	2.8	4.4	2.2	3.8	2.9	4.6	2.1	4.0	1.5	4.1	2.8	4.3	2.2	4.3	2.9	4.4	2.5	4.5	2.7	4.4	2.5	4.6	2.7	4.3	2.2	4.4

*3D, three-dimension; S3D, stereoscopic 3D; HP, healthcare professionals*.

## Discussion

The present work illustrates novel applications of stereoscopy for visualizing brain data from leading magnetic resonance imaging modalities. These preliminary examples convey the ability of 3D visualization approaches to better communicate the complexities and dimensionality of human brain structure and function. In particular, our functional connectivity atlas based on the Craddock et al. (2012) parcellation demonstrates the added value of stereoscopic approaches in communicating network relationships, as they successfully convey information regarding the relative position of network components typically lost in two-dimensional representations. Additionally, our work suggests possible clinical and educational value for the visualization of lesions (e.g., tumors, plaques) in the brain.

While a variety of approaches are available to create 3D visualizations, the present work used the popular anaglyph method (using red-cyan glasses). This approach is advantageous due to the relative ease and low cost by which 3D visualization can be achieved, simply requiring red-cyan glasses that are commonly available and can be purchased for as little as US $0.50. Of course, visualization without the need for glasses would be optimal, though is unlikely to be obtained without incurring significantly greater expense (e.g., $19,999 for a glasses-free 3D TV with lenticular lens). Importantly, anaglyph images can be generated with packages such as 3D Slicer and displayed on a television screen, computer screen, cinema, or printed in a manuscript or poster. The selection of red-cyan as opposed to other possible color combinations for generation of anaglyphs leaves researchers an ample array of color options for use in their images. Some caution does need to be taken in the selection of colors for use in the generation of anaglyph images, which can be somewhat time-consuming, although given the additional benefits in the final results, careful selection is merited.

The stereoscopic functional connectivity atlas is useful for viewing the relative position of the different parts of each of the 200 functional connectivity networks, including the relative position of the functional networks with respect to subcortical structures and, among the different networks themselves. SAIBN could be also useful for visual comparison of functional connectivity networks corresponding to an individual patient in reference to the 200 atlas networks, as well as for educational purposes.

Importantly, stereoscopy may not be an ideal solution for all visualizations. Specifically, some individuals may have problems with 3D perception. Stereoblindness is the complete deficiency of stereopsis, and is often caused by strabismus (crossed-eye), amblyopia (lazy eye), or blindness in one eye (Lipton, 1982). Lipton (1982) specified that 2% of people are stereo-blind, and another 2% or 3% of healthy individuals suffer fatigue and discomfort when looking at stereo projections.

Additionally, Sherman (1953) found that approximately 2% of the population has hyperphoria, an upward deviation of the visual axis of one eye relative to the other, which prevents the visual axes of the eyes from crossing at any point in space and hence precludes stereoscopy. Total or partial color-blindness can also produce some limitations for viewing in 3D using anaglyph-based methods or other similar ones, and has a prevalence of 1.3% (Lipton, 1982; Pokorny and Smith, 1986). Beyond these various conditions, additional considerations are the potential discomfort of wearing 3D stereoscopic glasses for those that do not wear glasses regularly, and the need to overlay them over corrective lenses for those who do wear glasses.

Our evaluation shows that a diverse array of healthcare professionals that explored the 3D and stereoscopic images perceived the increased dimensionality of stereoscopy as beneficial. Depth of tumors, relative position of MS lesions, relative position, and size of both hippocampi, and relative position of different functional connectivity areas in resting state images were better perceived in stereoscopic images than in common 3D images (percentage difference greater than 30%). Higher percentage difference was obtained for the tumor related images (more that 98%) and lower percentage difference (lower than 20%) for images that include a functional area in close contact with a tumor (for example Figure 7).

In conclusion, the present work illustrates the potential utility of 3D stereoscopic visualization approaches in visualizing complex imaging findings. Using several imaging approaches (e.g., task activation fMRI, fMRI-based functional connectivity, morphometry, and tissue segmentation) and findings (e.g., cyst, tumor, multiple sclerosis), we demonstrated the ease with which stereoscopy can be applied to imaging data to provide more realistic and informative representations of brain structure and function. In particular, stereoscopy uniquely provided intuitive descriptions of the exact location and the relative size of various normal elements and lesions in a 3D representation, and facilitated comprehension of the anatomical position of complex large-scale functional connectivity patterns. Novel 3D visualization techniques can enhance the utility of imaging data for clinical applications and offer, through their intuitive nature, substantial educational value. Future work will benefit from further quantitative evaluation of the benefits of 3D stereoscopic visualization of neuroimages.

### Conflict of interest statement

The authors declare that the research was conducted in the absence of any commercial or financial relationships that could be construed as a potential conflict of interest.

## Supplementary material

Supplementary Video 1 **Spinning stereoscopic (anaglyph) video of Patient P3 pial surface (epilepsy secondary to right precentral tumor, 36 years old)**. Must be viewed using red-cyan anaglyph glasses.

Supplementary Video 2 **Spinning stereoscopic (anaglyph) video of Patient P5 (medulla oblongata cavernoma, 63 years old)**. Corticospinal tract with cavernoma in yellow.

Supplementary Video 3 **Spinning stereoscopic (anaglyph) video of lesion load of patient P8 (multiple sclerosis, 42 year-old male)**.

Supplementary Video 4 **Spinning stereoscopic (anaglyph) version of Default Mode Network obtained using resting state images from 45 controls**.

## Abbreviations

ICN: intrinsic connectivity networks
MRI: magnetic resonance imaging
MS: multiple sclerosis
PET: positron emission tomography
SPECT: single-photon emission computed tomography, fMRI, functional magnetic resonance imaging
DTI: diffusion tensor imaging
FLAIR: fluid attenuated inversion recovery.
